# The Oncological Follow-Up of Fertility Sparing Surgery for Mucinous Borderline Ovarian Tumours: A Retrospective Cohort Study

**DOI:** 10.3390/cancers17233825

**Published:** 2025-11-28

**Authors:** Nicholas Anson, Patricia Cox, Lorraine S. Kasaven, Chiara Landolfo, Maya Al-Memar, Benjamin P. Jones, Srdjan Saso, Mona El-Bahrawy, Sadaf Ghaem-Maghami, Joseph Yazbek

**Affiliations:** 1 Division of Surgery and Cancer, Institute of Reproductive & Developmental Biology, Imperial College London, Hammersmith Hospital Campus, Du Cane Road, London W12 0NN, UK; l.kasaven@nhs.net (L.S.K.); srdjan.saso@nhs.net (S.S.); s.ghaem-maghami@imperial.ac.uk (S.G.-M.); joseph.yazbek@nhs.net (J.Y.); 2 Queen Charlotte’s and Chelsea Hospital, Imperial College Healthcare NHS Trust, London W12 0HS, UK; patricia.cox6@nhs.net (P.C.); chiara.landolfo@nhs.net (C.L.); maya.almemar@nhs.net (M.A.-M.); benjamin.jones@nhs.net (B.P.J.); 3 Department of Metabolism, Digestion & Reproduction, Faculty of Medicine, Imperial College London, London W2 1NY, UK; 4 The Lister Fertility Clinic, The Lister Hospital, Chelsea Bridge Road, London SW1W 8RH, UK; 5 Department of Metabolism, Digestion and Reproduction, Imperial College London, Hammersmith Hospital Campus, Du Cane Road, London W12 0NN, UK; m.elbahrawy@imperial.ac.uk

**Keywords:** mucinous borderline ovarian tumour, fertility sparing surgery, oncological follow-up, unilateral salpingo-oophorectomy, ovarian cystectomy

## Abstract

**Background/Objectives**: To determine whether long-term oncological follow-up is required following fertility-sparing surgery (FSS) for mucinous borderline ovarian tumours (MBOTs). **Methods**: A retrospective cohort study set in the tertiary gynaecology oncology centre at Imperial College Healthcare NHS Trust. Patients included were those under follow-up post-surgery for an MBOT in the ovarian clinic from 2007 to 2025. Rate of recurrence was compared amongst patients who underwent ovarian cystectomy, unilateral salpingo-oophorectomy (USO) or bilateral salpingo-oophorectomy (BSO) +/− hysterectomy. **Results**: From 74 patients diagnosed with MBOT, 36.5% (27/74) underwent BSO +/− hysterectomy and 63.5% (47/74) had FSS. Of the patients who underwent FSS, 59.6% (28/47) had an initial USO and 40.4% (19/47) underwent ovarian cystectomy. Subsequently, 63.2% (12/19) of patients who initially had ovarian cystectomy proceeded with completion USO, leading to a total of 40 USOs performed. There were no recurrences following BSO +/− hysterectomy, primary USO or completion USO after a median follow-up of 49.0, 65.5 and 48.0 months, respectively. Of the patients who underwent ovarian cystectomy, 15.8% (3/19) were found to have residual MBOT (n = 1) two months post-cystectomy or MBOT recurrence (n = 2) at 10- and 66-months post-cystectomy, all diagnosed at USO. There is a significant association between ovarian cystectomy and disease recurrence (Fisher’s exact test *p* = 0.015). **Conclusions**: Patients of reproductive age who undergo USO for a MBOT can be offered a reduced follow-up schedule as the risk of recurrence is very low. In contrast, patients who are managed by ovarian cystectomy have a higher risk of recurrence and require long-term surveillance monitoring.

## Simple Summary

Young women with suspected mucinous borderline ovarian tumours, a type of ovarian cyst, require surgery. Due to the large size and nature of these lesions, removal of the entire ovary and fallopian tube is typically required and recommended; however, some patients undergo surgery to remove the cyst alone. Following surgery, patients undergo long-term follow-up with ultrasound imaging as surveillance for disease recurrence. We set out to determine if this long-term follow-up is required in all patients. Our results indicate that, in patients who have undergone removal of the ovary and fallopian tube, the risk of recurrence is very low and therefore we suggest these patients can be offered a reduced follow-up schedule. For patients treated with removal of the cyst alone, there is a higher chance of disease recurrence and therefore these patients should undergo long-term follow-up as is currently practiced.

## 1. Introduction

There are six histological types of borderline ovarian tumours (BOTs), categorised by epithelial cell differentiation. The most common are serous (SBOT), followed by mucinous (MBOT), comprising approximately 50% and 45% of diagnoses, respectively. Endometrioid, clear cell, seromucinous (previously endo-cervical type mucinous) and Brenner BOT comprise the remainder. Common to all types is the absence of invasive malignancy [1,2,3,4,5].

MBOTs are non-invasive mucinous neoplasms with complex architecture and gastrointestinal-type differentiation [1,2,6]. Over 90% are unilateral and are often characterised by their large size of up to 50 cm and distinct radiographic appearances, such as a honeycomb nodule and the presence of over 10 locules [7,8,9,10]. Given the relatively high recurrence for MBOTs treated with ovarian cystectomy, it is typically recommended that patients with a presumed diagnosis of a unilateral MBOT undergo unilateral salpingo-oophorectomy (USO) [11,12,13].

The 2019 European consensus guidelines advised that in a patient diagnosed with an MBOT, disease found outside of the ovary should be considered a metastasis from an undiagnosed ovarian carcinoma [11]. Alternatively, lesions found within the ovary or extraovarian, may present as metastases from a primary mucinous carcinoma elsewhere, typically the gastro-intestinal tract [11,14].

In light of excellent survival rates for BOTs, there has been an evolution in surgical management, with fertility-sparing surgery (FSS) now routinely offered to women of reproductive age [4,13,15,16]. FSS is defined as the preservation of the uterus and at least one part of one ovary [15]. All patients undergoing FSS are advised to have long-term follow-up with interval imaging and clinical assessment to ensure early recognition of disease recurrence [4,17].

Recent guidance advises that patients with BOTs can attempt spontaneous conception immediately following FSS and in the case of early stage disease, patients can undergo immediate post-operative assessment for fertility treatment [15]. In a cohort of 172 patients undergoing FSS for BOTs, 55 pregnancies were achieved, resulting in 37 livebirths [16]. Furthermore, routine completion surgery, defined as the removal of the remaining ovary and tube, is no longer recommended in women who have undergone FSS [15]. In post-menopausal patients, bilateral salpingo-oophorectomy with or without hysterectomy remains the standard management of BOTs [11].

Considering recent changes to the recommendations regarding the histological diagnosis and surgical management of unilateral MBOTs, we aim to determine a suitable follow-up strategy for patients diagnosed with unilateral stage 1 MBOT (International Federation of Gynaecology and Obstetrics (FIGO) 2021) who have undergone FSS with full surgical staging [18]. These results can be used to standardise MBOT management guidelines.

## 2. Materials and Methods

This is a retrospective cohort study of all patients diagnosed and treated for a MBOT (Somerset Cancer Register, National Health Service, UK) who underwent assessment and follow-up in the tertiary ovarian clinic at Queen Charlotte’s and Chelsea Hospital (QCCH) between 2007 and 2025 inclusive. Some patients underwent initial surgery at their local hospital, with their care transferred to QCCH following histological confirmation of MBOT.

There were 27 patients who underwent bilateral salpingo-oophorectomy (BSO) and staging, 23 of whom also had a hysterectomy performed. Forty-seven patients underwent FSS, which includes (i) 28 USO and staging procedures and (ii) 19 ovarian cystectomies (Table 1). Subsequently, 12 patients treated with ovarian cystectomy went on to have a completion USO. As such, a total of 86 procedures were performed on 74 patients. All patients treated with BSO +/− hysterectomy or USO underwent full radiological, histopathological and surgical staging of disease. Full surgical staging is defined as visual assessment of the abdomen and pelvis, including the appendix with removal if abnormal, and targeted peritoneal and omental biopsies. Twelve of the nineteen patients who underwent ovarian cystectomy were not suspected to have an MBOT pre-operatively, and therefore full surgical staging was not performed at the time of the index surgery.

Subsequent follow-up was provided at a dedicated consultant-led ovarian cyst clinic, with tertiary level expertise to optimise the management of patients with ovarian cysts. All patients underwent expert ultrasound assessment at each appointment. Some patients had their follow-up care transferred to units outside of the Imperial College Healthcare Trust (ICHT), typically back to the initial referring unit and are included in the analysis as there was full access to their medical records. Women were deemed to have sufficient follow-up to be included in this analysis if they remained under regular follow-up or were discharged after a prolonged course of follow-up without recurrence, typically at five years. One patient requested discharge 22 months post-BSO and hysterectomy, and another died 27 months post-BSO and hysterectomy in the absence of disease recurrence, both of whom are included in this analysis.

The main outcomes measured were as follows: type of surgery performed, duration of follow-up, recurrence rate of disease and subsequent surgical management of primary or secondary disease. Secondary outcome measures included age at index surgery, FIGO staging and surgical technique (laparotomy vs. laparoscopy). The duration of follow-up was calculated to the date of the patient’s most recent clinic attendance. Statistical analysis was conducted with IBM SPSS Statistics for Windows, v28 (IBM Corp., Armonk, NY, USA) with statistical techniques described in the text.

## 3. Results

### 3.1. Subsection

#### 3.1.1. Patient Cohort

**Figure 1:**
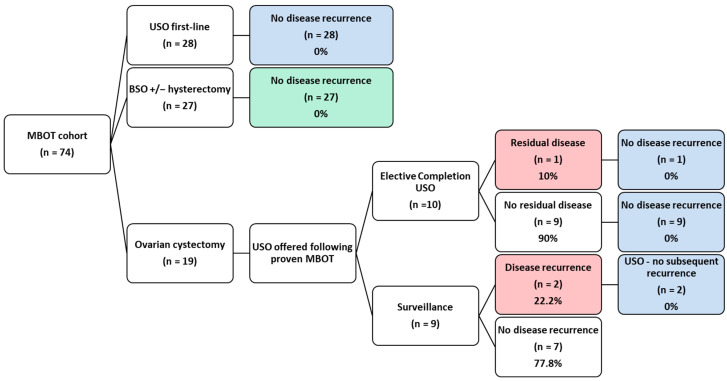
Follow-up flow chart. Rates of residual and recurrent MBOT, and the follow-up of patients who underwent all types of surgery, including the specific details for the follow-up of patients treated with initial ovarian cystectomy. A total of 86 procedures were performed on 74 patients as 12 patients underwent USO after an initial cystectomy. Blue = no disease recurrence following USO; Green = no disease recurrence following BSO +/− hysterectomy; Red = residual or recurrent disease.

There were 74 patients diagnosed with MBOT who underwent surgical management between 2007 and 2025, of which 36.5% (27/74) had BSO +/− hysterectomy and 63.5% (47/74) underwent FSS. In total, 19 ovarian cystectomies, 40 USO and 27 BSO +/− hysterectomy procedures were performed (Table 1). Twelve women who underwent ovarian cystectomy subsequently underwent a USO, either for elective completion or suspicion of recurrence, as detailed in Figure 1. All patients had unilateral disease deemed to be FIGO stage 1 by a post-operative multi-disciplinary team (MDT) meeting consisting of gynae-oncologists, histopathologists and radiologists.

#### 3.1.2. Bilateral Salpingo-Oophorectomy +/− Hysterectomy

Of these 27 patients, 23 underwent BSO and hysterectomy, and four underwent BSO alone. The median age at surgery was 62 years (IQR 52.0–72.5) (Table 2). Eight patients were discharged after a median 56.5 months (IQR 51.3–67.0) follow-up with interval ultrasonography. A further 18 patients remain under active follow-up (Table 1). Amongst the cohort, 85.2% (n = 23) underwent laparotomy and 14.8% (n = 4) underwent laparoscopy (Table 2). There were no recurrences of MBOT in this group.

#### 3.1.3. Unilateral Salpingo-Oophorectomy

Of the 40 patients who underwent USO, 28 underwent primary USO, while 12 had USO following MBOT diagnosis at initial ovarian cystectomy (Figure 1). There were no recurrences of MBOT amongst the 40 patients who underwent USO, which includes the three patients with residual or recurrent disease following cystectomy.

There was no significant difference in median age between patients who underwent primary USO (31.5, IQR 26.8–36.3) or USO after cystectomy (30.0, IQR 28.8–36.3), as assessed by a Mann–Whitney U test (*p* = 0.965). The surgical approach differed significantly between groups (Fisher’s exact test, *p* < 0.001). In the primary USO group, 96.4% (27/28) underwent laparotomy vs. 3.6% (1/28) who had laparoscopy. In the USO after cystectomy group, 75.0% (9/12) underwent laparoscopy vs. 25.0% (3/12) who had laparotomy. Disease staging also differed significantly between groups (Fisher’s exact test, *p* = 0.011). In the primary USO group, 78.6% (22/28) were classified as FIGO 1A vs. 21.4% (6/28) as FIGO 1C. In the USO after cystectomy group, 33.3% (4/12) were classified as FIGO 1A vs. 66.6% (8/12) as FIGO 1C. While the median time under follow-up after USO was longer for patients who had primary USO, rather than USO after initial cystectomy (65.5 months, IQR 39.0–110.3 vs. 48.0 months, IQR 21.3–132.8), this was not statistically significant (Mann–Whitney U Test *p* = 0.805).

#### 3.1.4. Ovarian Cystectomy

The 19 patients who underwent ovarian cystectomy include 7 patients who had a differential diagnosis of an MBOT prior to surgery and 12 who had MBOT diagnosed following surgery. As such, not all cystectomy patients underwent full staging at the time of their initial surgery. The median age at cystectomy was 30.0 years (IQR 24.5–32.5).

For the seven patients who underwent cystectomy only, the median duration of follow-up was 133.0 months (IQR 78.0–149.0). The 12 patients who underwent subsequent USO had a median follow-up of 2.0 months (IQR 1.8–5.5) prior to USO, as most patients elected for completion USO shortly after receiving a diagnosis of MBOT.

All cystectomy patients who received a diagnosis of MBOT were offered USO. Ten patients chose to proceed with USO, while nine elected for ultrasound surveillance. Overall, 15.8% (3/19) of patients were found to have disease recurrence (n = 2) or residual MBOT (n = 1). Amongst the patients who requested ultrasound surveillance, the recurrence rate was 22.2% (2/9), as seen in Figure 1.

The two patients with sonographic evidence of recurrence underwent USO at 10 and 66 months after their initial cystectomy. The patient operated on at 66 months post-cystectomy had not been seen in clinic for two years before recurrence, as she was abroad. The patient with residual MBOT in the remaining ovarian tissue was diagnosed when she underwent elective completion USO two months post-cystectomy.

**Figure 2:**
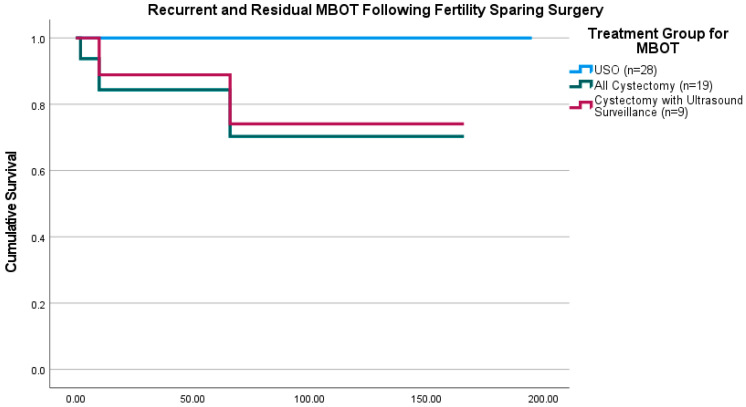
Kaplan–Meier curves for disease-free survival following fertility-sparing surgery in patients with MBOTs. The blue line represents the USO group (n = 28; 0 recurrences), the green line represents all patients who underwent ovarian cystectomy group (n = 19; 1 residual and 2 recurrences) and the purple line represents the subset of cystectomy patients who opted for ultrasound surveillance (n = 9; 2 recurrences). A log-rank test indicated statistically significant differences between the USO and all cystectomy groups (*p* = 0.011) and between the USO and cystectomy with ultrasound surveillance groups (*p* = 0.026).

Fisher’s exact test was used to compare recurrent and residual disease amongst treatment groups following initial surgery. This found a significant association between ovarian cystectomy and recurrent or residual disease (*p* = 0.015). Figure 2 displays the Kaplan–Meier cumulative disease-free survival comparison for patients who underwent FSS. This divides the cystectomy group into all cystectomy patients and those who opted for ultrasound surveillance, with both groups significantly more likely to suffer disease recurrence that the USO group.

## 4. Discussion

### 4.1. Summary of Findings

Our data supports the recommendation that USO with surgical staging is a safe and potentially definitive treatment for unilateral stage 1 MBOTs in pre-menopausal women. However, women treated with ovarian cystectomy for MBOTs are at increased risk of recurrence, which remains consistent with the published literature [19,20].

**Figure 3:**
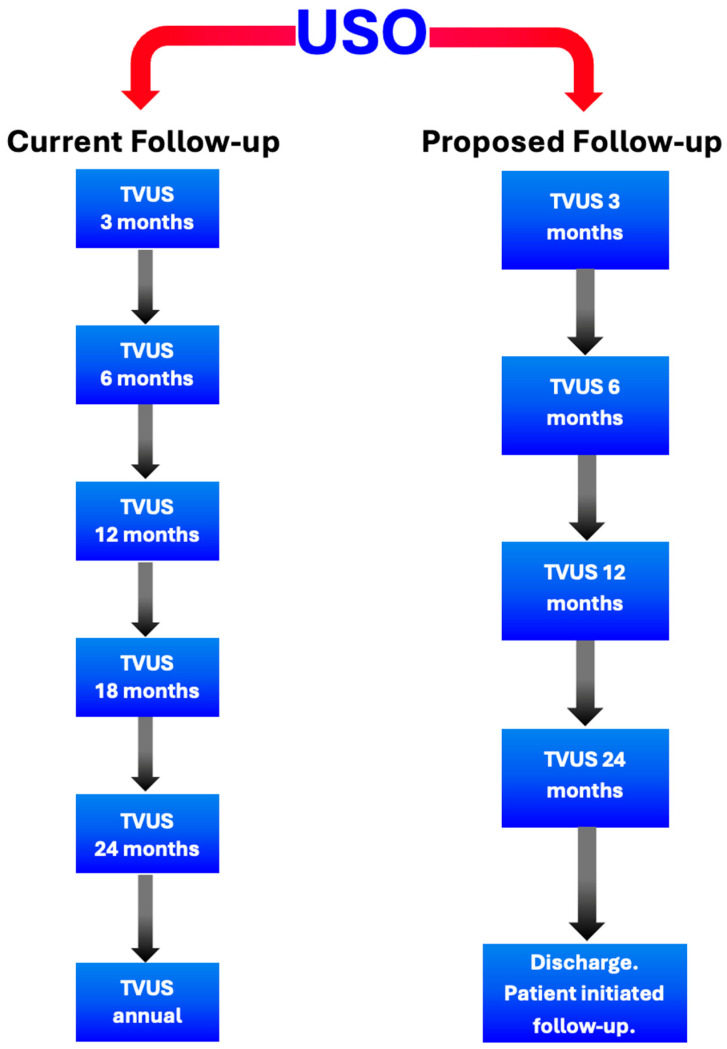
Current and proposed follow-up of MBOTs following USO.

While national guidance suggests that long-term follow-up beyond five years is required post-operatively for women with BOTs, this is not specific to histological subtype [4]. Given our recurrence rate of 0.0%, we propose that women treated with USO and full staging who receive a diagnosis of stage 1 MBOT can undergo less-intensive follow-up after surgery, with no subsequent impact on mortality (Figure 3). However, ovarian or extraovarian mucinous carcinoma should be excluded prior to discharge. We propose discharge 24 months following surgery if ultrasound is normal, with patient-initiated follow-up available in case of symptoms suggestive of recurrence. For women who desire prolonged follow-up with interval sonography, an annual scan is offered for up to five years.

A significantly reduced follow-up schedule offers substantial benefits for both patients and healthcare providers, including cost savings from fewer outpatient appointments and reduced time off work. Equally important are the potential psychological advantages; women with BOT often perceive their condition as having a malignant potential comparable to invasive cancer rather than a benign ovarian cyst [21]. The proposed follow-up approach, coupled with enhanced communication, may help mitigate these perceptions.

### 4.2. Psychological Impact of Reduced Follow-Up

A meta-analysis assessing both the European Organisation for the Research and Treatment of Cancer Quality of Life Questionnaire and the Hospital Anxiety and Depression Scale found non-inferiority of low-intensity follow-up after oncological surgery [22]. The study covers multiple cancer types and variations in follow-up practice from doctor-led, to nurse-led and patient-initiated. There was no significant difference in physical, role, emotional, cognitive and social functioning, global health status, fatigue, anxiety and depression when patients underwent low-intensity follow-up.

A review assessing quality of life following treatment for gynaecological cancers found that patients perceived the primary focus of routine follow-up as the early detection of relapse [23]. When patients are informed of the lack of evidence for routine follow-up, the majority are less likely to desire this [23]. Data specific to the psychological impact of follow-up in BOT patients is limited. Studies show women with BOTs have a good quality of life following treatment, which is comparable to that of the general population [24,25]. Conversely, one small study found generally high anxiety levels in both BOT and benign patients, with BOT patients likely to suffer from a higher burden of disease and treatment [26]. Our proposed reduced follow-up schedule, supported by the low risk of recurrence of MBOT following USO, may help reduce psychological sequelae in women with MBOTs.

### 4.3. Safety of Reduced Follow-Up

Regarding the safety of early discharge, we compare our findings to the literature with respect to previous clinical recommendations, including the changes in diagnostic criteria for MBOTs. Primarily, this has entailed full surgical staging, exclusion of seromucinous BOT and histopathological measures to rule out occult mucinous carcinoma. As such, earlier studies have demonstrated heterogeneity in the reporting of recurrence rates for patients who have undergone USO for MBOTs [19,27,28]. Despite this, the recurrence rate for MBOTs treated with USO is consistently reported as significantly lower than for SBOTs and for MBOT patients treated with ovarian cystectomy [19,27,28].

Fertility preservation surgery for patients with BOTs should not preclude complete staging. The 2024 guidance from the British Gynaecological Cancer Society states that all surgery for BOTs should include peritoneal staging with inspection, washings for cytology and peritoneal biopsies, omental biopsy or omentectomy, and visual assessment of the appendix with subsequent removal if macroscopically abnormal [4]. If this is undertaken, in combination with the advised histopathological assessment, then it is shown that patients treated with USO have a low risk of recurrence [29].

A 2017 single-centre retrospective study of 81 patients diagnosed and treated for unilateral intestinal-type MBOTs over 10 years (all <FIGO stage II) found no recurrences in the 14 patients who underwent USO with staging and expert histopathological assessment over a median follow-up of 87 months [29]. Similarly to our findings, the study demonstrates favourable outcomes amongst patients undergoing fertility-sparing surgery with USO. Other recent publications report on smaller MBOT cohorts without evidence of recurrence following FSS; however, the exact surgical procedure undertaken is not always clear [30,31,32].

Conversely, earlier studies have demonstrated higher recurrence rates and lower disease-free survival for MBOTs [19,33]. As an example, in one retrospective series of 100 patients who underwent FSS, there were 5 recurrences amongst 20 patients who underwent cystectomy (two invasive) and 10 recurrences amongst 80 patients who underwent USO (six invasive) [12]. However, this study has several limitations, including that not all patients underwent standardised initial surgery and histopathological assessment prior to referral to a tertiary centre, and omental sampling and peritoneal assessments (cytology or biopsy) at the time of initial surgery were not performed in all patients. Consequently, the authors accepted the possibility of a small focus of invasive carcinoma having been missed at initial assessment on account of the typically large size of MBOTs.

A 2001 prospective study of 339 women with both SBOTs and MBOTs reported a disease-free survival rate of 99.3% in stage 1 patients treated conservatively [34]. Of these, 189 patients underwent fertility-sparing surgery and 3.3% of MBOTs (4 out of 117) treated with USO or cystectomy recurred. Two patients with MBOTs treated with cystectomy progressed to carcinoma. As this study was published in 2001, the diagnostic criteria and staging procedures for MBOTs undertaken will differ from those in our cohort.

In other cohorts where recurrence for MBOT patients who have undergone FSS is reported on, procedure-specific data are often missing, making a direct comparison with our data potentially inaccurate [19,27,28]. In the small number of cases where recurrence following USO for MBOTs is noted in more recent publications, the data are often not recent or the methodology for surgical staging and histopathological assessment is reported as potentially deficient or is not described [35,36,37,38]. Included in a large recent cohort looking exclusively at 232 MBOT patients are 71 patients treated with USO, one of whom recurred 7 months post-surgery outside of the abdomen; however, the staging undertaken may have differed from the standard at our institution [39].

If cystectomy is performed for a suspected MBOT, it is essential that full staging is performed at the time of initial surgery [4]. These patients should be offered USO in the first instance, or indefinite ultrasound surveillance. As demonstrated by the patients in our dataset, cystectomy does have an increased recurrence rate and therefore patients who have undergone cystectomy only should not be discharged.

Guidance states that the sampling of MBOTs should occur at a minimum of one block per cm of the tumour’s maximum dimension. If there are features such as microinvasion or intraepithelial carcinoma, or a tumour size over 10 cm, then two blocks per cm of the tumour’s maximum dimension should be assessed [2,4,5,40]. Mucinous tumours are noted to have a greater heterogeneity of histology within a lesion, thereby necessitating thorough assessment to rule out foci of invasive carcinoma [5,41].

Some patients are diagnosed with MBOTs incidentally, following an ovarian cystectomy or USO in the absence of full staging surgery, including assessment of the appendix. These patients should be counselled as to the potential benefits of ipsilateral oophorectomy or a restaging procedure, respectively [4,42].

### 4.4. Strength and Limitations

This study’s main strengths are the large patient cohort and consistency in MBOT management at a single tertiary gynaecological oncology centre throughout the assessed period. The primary limitation of this study is its retrospective nature. Given the absence of high-quality prospective studies on BOTs, particularly since recent changes to the WHO histopathological guidelines, such data would add significant weight to the proposed recommendations in this study. Any future work should clearly define the staging and diagnostic criteria used, with the exact procedures patients underwent clearly stated, especially in the case of recurrence. Studies with a longer duration of follow-up would help demonstrate that USO is a potentially definitive treatment.

## 5. Conclusions

Our data suggest that in pre-menopausal patients who have undergone USO and full staging for unilateral stage 1 MBOTs, the frequency and length of follow-up can be safely reduced. We propose discharge 24 months following USO in the context normal surveillance imaging. Patient-initiated follow-up for any symptomatic concerns should be organised. If patients desire continued follow-up, an annual scan for up to five years can be offered.

Patients who undergo ovarian cystectomy for MBOTs should continue long-term follow-up with interval ultrasound monitoring as the risk of recurrence is significantly higher. We do not propose any changes to the follow-up of patients treated with ovarian cystectomy or that of post-menopausal patients treated with bilateral salpingo-oophorectomy with or without hysterectomy.

## Figures and Tables

**Table 1:** Operations performed and patient follow-up status. A total of 86 procedures were performed on 74 patients as 12 patients underwent USO after an initial cystectomy.

	Number (n)	Discharged After Sufficient Follow-Up	Ongoing Follow-Up	Recurrence/Residual	Deceased Included
**BSO +/− hysterectomy**	27	8	18	0	1 *
**USO**	28	0	27	0	1 ^+^
**USO after cystectomy**	12	0	12	0	0
**Ovarian cystectomy**	19	0	19	3	0

* Patient died from unrelated illness with 27 months of follow-up data. ^+^ Patient died from unrelated illness with 63 months of follow-up data.

**Table 2:** Patient demographics, time under follow-up, tumour size, surgical details and disease staging.

	BSO +/− Hysterectomy	USO	USO After Cystectomy	Ovarian Cystectomy	
Median Age at Surgery (IQR)	62.0 (52.0–72.5)	31.5 (26.8–36.3)	30.0 (28.8–36.3)	30.0 (24.5–32.5)	
Age range	39–80	22–46	18–46	18–45	
Median months in FU (IQR)	49.0 (19.0–67.0)	65.5 (39.0–110.3)	48.0 (21.3–132.8)	Cystectomy Only	Subsequent USO
				133.0 (78.0–149.0)	2.0 (1.8–5.5)
Range of months in FU	4–128	4–195	6–189	Cystectomy Only	Subsequent USO
				47–166	0–66
Median Largest Dimension mm (IQR)	177.0 * (151.3–227.5)	202.5 (159.0–302.5)	NR **	130.0 (100.0–168.5)	
Laparoscopy	14.8% (4/27)	3.6% (1/28)	75.0% (9/12)	42.1% (8/19)	
Laparotomy	85.2% (23/27)	96.4% (27/28)	25.0% (3/12)	57.9% (11/19)	
FIGO 1A	80.8% (22/27)	78.6% (22/28)	33.3% (4/12)	42.1% (8/19)	
FIGO 1C	19.2% (5/27)	21.4% (6/28)	66.6% (8/12)	57.9% (11/19)	
Pre-menopausal	25.9% (7/27)	100% (28/28)	100% (12/12)	100% (19/19)	
Post-menopausal	74.1% (20/27)	0% (0/28)	0% (0/12)	0% (0/19)	
Left sided	51.9% (14/27)	53.6% (15/28)	66.6% (8/12)	73.7% (14/19)	
Right sided	48.1% (13/27)	46.4% (13/28)	33.3% (4/12)	26.3% (5/19)	

* Pre-operative tumour size could not be determined for one patient undergoing BSO. ** Tumour size is not recorded as most patients underwent completion surgery without an ovarian lesion present.

## Data Availability

The data used in the study are available upon request from the authors.

## Abbreviations

The following abbreviations are used in this manuscript:

MBOT	Mucinous borderline ovarian tumour
SBOT	Serous borderline ovarian tumour
USO	Unilateral salpingo-oophorectomy
BSO	Bilateral salpingo-oophorectomy
FSS	Fertility-sparing surgery
TVUS	Transvaginal ultrasound scan
IQR	Inter-quartile range
FIGO	International Federation of Gynaecology and Obstetrics
QCCH	Queen Charlotte’s and Chelsea Hospital
ICHT	Imperial College Healthcare Trust
